# Gastrointestinal Involvement in Anderson-Fabry Disease: A Narrative Review

**DOI:** 10.3390/ijerph18063320

**Published:** 2021-03-23

**Authors:** Fabio Caputo, Lisa Lungaro, Adriana Galdi, Eleonora Zoli, Fiorella Giancola, Giacomo Caio, Roberto De Giorgio, Giorgio Zoli

**Affiliations:** 1 Department of Translational Medicine, University of Ferrara, 44121 Ferrara, Italy; lisa.lungaro@unife.it (L.L.); caigmp@unife.it (G.C.); dgrrrt@unife.it (R.D.G.); giorgio.zoli@unife.it (G.Z.); 2 Department of Internal Medicine, S.S. Annunziata Hospital, Cento (Ferrara), University of Ferrara, 44042 Ferrara, Italy; a.galdi@ausl.fe.it (A.G.); Zolieleonora@yahoo.com (E.Z.); 3 Department of Veterinary Medical Sciences (UNI EN ISO 9001:2008), Alma Mater Studiorum University of Bologna, 40064 Bologna, Italy; fiorella.giancola2@unibo.it; 4 Mucosal Immunology and Biology Research Center, Massachusetts General Hospital-Harvard Medical School, Boston, MA 02114, USA

**Keywords:** fabry disease, gastrointestinal manifestation, ERT, Irritable Bowel Syndrome (IBS) like symptoms

## Abstract

Anderson-Fabry disease (FD) is an X-linked lysosomal storage disorder leading to a wide array of clinical manifestations. Among these, gastrointestinal (GI) symptoms such as abdominal pain, bloating, and diarrhea affect about half of the FD adults and more than half of FD children. GI symptoms could be the first manifestation of FD; however, being non-specific, they overlap with the clinical picture of other conditions, such as irritable bowel syndrome and inflammatory bowel disease. This common overlap is the main reason why FD patients are often unrecognized and diagnosis is delayed for many years. The present narrative review is aimed to promote awareness of the GI manifestations of FD amongst general practitioners and specialists and highlight the latest findings of this rare condition including diagnostic tools and therapies. Finally, we will discuss some preliminary data on a patient presenting with GI symptoms who turned to be affected by a variant of uncertain significance of alpha-galactosidase (GLA) gene.

## 1. Introduction

Anderson-Fabry disease (FD) (Online Mendelian Inheritance in Man [OMIM] #301500) is an X-linked lysosomal storage disorder caused by the mutations of the alpha-galactosidase (GLA) gene, leading to the galactosidase A (α-Gal A) deficiency. The lack of this enzyme promotes the accumulation of globotriaosylceramide (GL3) and other glycosphingolipids within lysosomes [1,2,3,4]. Thus, the intracellular accumulation of glycosphingolipids affects a variety of tissues and organs [5,6], including the kidneys, heart, and brain [7,8,9], and increases the possibility of developing ischemic stroke, small-fiber peripheral neuropathy, cardiac dysfunction, and chronic kidney disease, with the risk of fatal complications [10,11].

The diagnosis of FD is established by: (1) reduced levels of α-Gal A activity in blood leukocytes, whole blood (dried blood spot), or in tissues; (2) increased plasma globotriaosylsphingosine (lyso-Gb3) concentration, and (3) disease-causing GLA gene mutation [12,13,14]. In men, FD is diagnosed when the activity of α-galactosidase A in peripheral blood leukocytes is absent or reduced. In heterozygous women, FD diagnosis is confirmed only after molecular genetic tests, as they can still show low or normal levels of α-Gal A activity due to genetic mosaicism, thus making α-Gal A activity measurement not completely reliable [15].

The available therapy for FD is based on enzyme replacement therapy (ERT), which can be administered intravenously every two weeks using recombinant agalsidase alfa (Replagal^®^) and agalsidase beta (Fabrazyme^®^) [16,17,18]. The administration of ERT delays organ damage resulting in an improved overall quality of life for patients and, although very expensive, the benefits of therapy overcome costs. Pharmacological chaperones represent another available therapy for the treatment of FD. These small molecules stabilize misfolded proteins, improving enzymatic activity and reducing substrate accumulation. Although they can be given by oral administration, pharmacological chaperons can be used only for FD cases related to missense mutations [19].

Despite ERT and pharmacological chaperons representing a proper treatment to delay the disease progression, these therapies may be ineffective in FD patients carrying specific genetic polymorphisms. Indeed, these subjects are unresponsive to treatments and display illness evolution, with renal, cardiovascular, and cerebrovascular involvement [20].

These features will be more extensively discussed in Section 5.

Albeit considered very rare, the increasing attention given to this illness has increased genetic screenings, and nowadays the FD occurrence in newborns is about 1:3000 in Europe [15]. From a clinical standpoint, FD patients may also show two phenotypes characterized by either classic or later onset. In the first case, FD occurs in childhood with a broad range of manifestations, such as chronic neuropathic pain, occasional severe pain crisis, hypohidrosis, skin abnormalities (angiokeratomas), corneal opacity (*cornea verticillata*), and gastrointestinal (GI) symptoms, i.e., bloating, diarrhea, abdominal pain [21]. During the third to fourth decade of life, FD patients with classic phenotype experience renal failure (proteinuria, glomerulosclerosis, chronic kidney disease), heart complications (myocardial fibrosis, left ventricular hypertrophy, cardiac conduction, and valvular defects), and neurologic impairment (such as transient ischemic attacks and strokes). A “late-onset” (or “non-classic”) occurs in the fourth to seventh decade of life in patients showing the same cardiac and renal abnormalities similar to those of the classic phenotype [22]. Patients affected by the classical phenotype show a marked deficiency up to the absence of α-Gal A activity; this promotes glycosphingolipids accumulation (in particular GL3) into lysosomes of many cell types [1,23,24]. Late-onset patients maintain variable levels of residual α-Gal A activity and show a more heterogeneous clinical presentation, ranging from the classical and severe phenotypes to the late-onset variants characterized by heart complications (e.g., left ventricular hypertrophy, arrhythmia) [24,25,26,27,28,29].

The disease manifestation also changes according to gender. In heterozygous females, the genetic mosaicism due to the random inactivation of one X-chromosome creates more cell lineages expressing different sets of X-linked genes. This feature evokes variable disease manifestations, ranging from asymptomatic to the classical presentation as in male patients [30].

GI symptoms are very common in FD, affecting up to 50% of female patients and almost 60% of untreated children [4,30,31,32], according to the Registry data from patients enrolled in the Fabry Outcome Survey. Symptoms reported by FD patients include abdominal pain, bloating, early satiety, intermittent/chronic diarrhea, constipation, recurrent nausea and vomiting, and low weight gain [30,32,33,34,35,36,37] that overlap with those observed in common disorders, such as irritable bowel syndrome (IBS) or inflammatory bowel disease. A timely diagnosis could improve and save a patient’s life; thus, it is crucial to promote knowledge about GI manifestations of FD among specialists. This narrative review will discuss the main GI manifestations related to FD. Then, we will report a clinical case of a patient affected by a variant of uncertain significance (VUS) GLA gene mutation and presenting with typical GI symptoms.

## 2. Literature Retrieval

The purpose of this narrative review is to discuss the GI symptoms in FD patients, describing the latest findings on therapies. Two independent authors (L.L. and F.C.) assessed PubMed, EMBASE, MEDLINE, and Science Direct databases in two independent literature searches, carrying out the literature search in a stepwise fashion, based on title and abstract. The authors searched for the following keywords, alone or in combination: “Fabry disease”, “gastrointestinal manifestation”, “gastrointestinal symptoms”, “enzyme replacement therapy”, “diet”, “nutrition”. We included articles fulfilling the following criteria: (1) describing the GI symptoms in FD; (2) published in the last ten years (January 2010–December 2020); (3) written in English; (4) available full text. We excluded Mesh terms. We retrieved 1006 abstracts from databases, and after a thorough screening, 120 abstracts were selected. Of these, 78 were excluded and 42 articles were considered eligible. In order to find other relevant articles, we also assessed the reference list of the collected papers.

## 3. Gastrointestinal Symptoms in FD: Clinical Features and Physiopathology

This section will describe the most common GI manifestations of FD and discuss the mechanisms leading to symptom generation. The onset of GI symptoms/signs depends on gender and age, with a higher prevalence in males than females and in younger over older subjects. Hopking et al. [35] analyzed GI symptoms in 352 pediatric patients enrolled in the Fabry Registry. Of these, 194 were males and 158 were females. Non-specific GI symptoms heralded the onset of FD in about 23% (45 out of 194) of male patients with a median age of 5 years and in about 11% (18 out of 158) of girls with a median age of 9 years. The most typical GI disturbs were abdominal pain (affecting up to 26.7% of overall patients) and diarrhea (19.3% of all patients) [35]. 

Hoffmann and collaborators [30] analyzed 342 patients enrolled in the Fabry Outcome Survey database (271 adults and 71 children; 139 males and 203 females), with documented GI symptoms. Constipation showed a gender prevalence since it was nearly twice in female (16.7%) vs. male patients (8.6%) followed by nausea and vomiting. Abdominal pain was usually reported in the mid and lower abdomen and described as “bloating”, “cramping”, or a “burning” sensation, superficial abdominal skin tenderness and discomfort, evoked by eating and worsened by stress [10,33]. Furthermore, diarrhea related to food intake occurred more frequently in males (26%) than females (17%) and in children (25%; median age of onset: 15.5 years) than in adults (19.2%) [30]. Moreover, nausea and vomiting were equally complained by both genders (13.8% males vs. 10.1% females), with a different incidence of nausea between children and adults (15.5% vs. 11.4%) [30].

Some FD patients, mainly females, describe constipation instead of diarrhea [31], whereas other FD patients report diarrhea alternating to constipation interrupted by periods of regular bowel movements, complicating the diagnosis and the management of FD [38]. GI symptoms hamper food intake leading some FD patients to lose body weight [10]; however, recent data do not appear to support these findings [30]. Notably, compared to controls, boys with FD show a lower body mass index, a feature that has not been reported in girls, as the severity of the disease is more male-gender dependent [35].

Other GI signs include hemorrhoids, that were reported only in adults, gastritis or ulcer and pancreatitis [30], chronic intestinal pseudo-obstruction [37,39], diverticular disease [10,37,40,41,42], postprandial fullness and early satiety (dyspepsia) [22], delayed gastric emptying [43], and bowel ischemia [38,39]. The burden of GI symptoms, especially diarrhea, affects the quality of life, as reported in surveys (EQ-5D) of adults and pediatric FD patients [30,33]. In children, FD does not involve kidneys and heart, whereas GI symptoms affect the routine life leading to school absenteeism [33]. Moreover, FD patients do not exhibit abnormalities of serum protein, albumin, folate, vitamin B12, calcium, and phosphate [33,38]. However, in some cases, some patients may show anemia due to underlying renal and cardiac impairment [44]. FD patients in an advanced stage suffer from heat intolerance, fatigue, and hypohidrosis. Furthermore, exposure to sun and/or heat aggravates neuropathic pain, one of the typical signs of FD. In this context, “Fabry crises” are defined as episodes of pain provoked by abrupt changes in body temperature, such as fever, or high environmental temperature. For this reason, FD patients limit physical exercise and exposure to sunlight (both precipitating factors), therefore increasing the risk osteoporosis due to a lower synthesis of vitamin D [45,46].

The origin of GI symptoms is complex and triggered by many factors. Intestinal motility alteration is hypothesized to be induced by neuropathic and myopathic changes [33]. These dysfunctions alter the muscle wall structure and function and promote, in some cases, diverticuli (along with progression to diverticulitis and renal complications) [37]. Abdominal pain is thought to originate from the GI tract ischemia enhanced by small-fiber neuropathy. The latter, per se, can affect peristalsis, thus causing intestinal stasis, responsible for small intestinal bacterial overgrowth, and consequent diarrhea [47]. Moreover, GL3 deposition in enteric neuron and vessels impairs the autonomic nervous system, causing hyperactive uncoordinated contractions and gut inflammation, which together can contribute to diarrhea. Another process that causes diarrhea in FD is an unbalanced entero-hepatic circulation of bile acids (BAs). BAs originate in the liver from cholesterol and are stored in gallbladder where they remain until a meal stimulates gallbladder contraction, triggering their release into the duodenum. Afterwards, BAs are reabsorbed by the distal ileum, and return to the liver through the portal circulation. A disruption of entero-hepatic circulation impairs BAs handling, leading to diarrhea via osmotic mechanisms. Factors triggering the BAs-induced diarrhea are ascribable to a deficiency in fibroblast growth factor 19, that inhibits the BAs synthesis, and to genetic variations [47,48,49]. Abnormalities of the enterohepatic circulation of BAs have been reported to play a role in GI symptom generation in FD patients. Di Martino et al. [49] investigated genetic variants potentially associated with GI symptoms in 49 FD patients. Nine single nucleotide polymorphisms mapped within four genes (ABCB11, SLCO1B1, NR1I3 and ABCC5), involved in BA export, detoxification, and uptake in the liver, with a higher susceptibility to develop GI symptoms compared to patients without polymorphisms.

The accumulation of GL3 in the endothelium and smooth muscle cells is responsible for many intestinal complications: ischemia resulting from a mechanical reduction of the gut lumen; altered metabolism via impaired cell signaling pathways; increase in muscular extracellular matrix proliferation and vascular remodeling [33]. Sphingolipid accumulation in the ganglion cells of the autonomic nervous system is also responsible for upper GI symptoms such as delayed gastric emptying [50]. Furthermore, the neuropathy seen in FD patients is similar to that detectable in diabetics, suggesting similar mechanism [36]. Overall, we can conclude that GI symptoms can be triggered by a number of pathogenetic factors.

## 4. Investigations of Gastrointestinal Involvement in FD 

GI manifestations could be attributed to FD using some specific examinations. Radiologic exams are suggested to investigate blood flow and gut motility, whereas the doppler scan is more appropriate for the diagnosis of ischemic-like symptoms such as abdominal pain, and angiography can assess the involvement of the underlying vascular tissue. Upper GI symptoms could be investigated with gastric emptying measurement, and abnormal bowel movements via colonic transit time [50]. Endoscopy of the GI tract is a useful tool to determine mucosal damage or inflammation; however, they are generally macroscopically normal. Bowel biopsy reveals the presence of GL3 deposition in neurons of villi; these appear enlarged [41,50], and detectable with luxol fast blue positive stain in blood vessels and muscle cells of the muscularis mucosa. Moreover, smooth muscle and ganglion cells can be visible with electron microscopy scan-presenting electrondense intralysosomal striped ‘zebra-like’ bodies [51]. When a thorough analysis of GI specimens will be possible, features usually found in dermal, cardiac, and renal tissues (i.e., vascular sclerosis and hypertrophy of smooth muscles, neuronal swelling and demyelination) will be probably identified in the GI tract [52]. In conclusion, many investigations can be performed to determine the GI involvement in patients with suspected FD, making easier and more immediate the identification of the illness.

## 5. Effects of Currently Available Therapies on GI Symptoms

This section will present the current available therapies, mostly ERT, for FD treatment, going from the most consolidated ones to the most recently available ones. We will also present data from studies investigating the effect of ERT on GI symptoms. The results of the studies discussed below are summarized in Table 1. 

ERT can provide a beneficial therapeutic approach to FD. However, much of the current treatments begin when substantial organ damage has already occurred. There is a need for updated and specific guidelines for adult patients, aimed to promote: (1) a personal approach to care, tailored on the specific genetic mutation and to the specific disease phenotype; (2) the prompt ERT initiation; (3) the routine follow-ups in order to monitor possible organ involvement in non-classic asymptomatic patients, and the response to therapy in treated patients; (4) the adjuvant treatments for specific disease manifestations, and (5) an experienced multidisciplinary team [23].

ERT comprises two recombinant human α-galactosidases enzymes, administered intravenously and available in Europe since 2001, named agalsidase alfa and agalsidase beta [53]. The cost of ERT therapy is very high, and it is estimated to be about 250,000 euro per year [54]. Overall, ERT has shown great effectiveness, providing benefits to organ systems and improving patients’ life expectancy.

A comprehensive systematic literature review on recombinant human α-galactosidase ERT clinical effects on both males and females show that it lowers GL3 levels in plasma and urine and skin cell types. ERT also decreases GL3 levels in organs such as kidney (retarding the glomerular filtration rate deterioration) and heart (stabilizing left ventricular mass and cardiac wall thickness). The therapy acts positively towards the nervous and GI system, improving pain and quality of life [16,55].

In addition, migalastat, a pharmacologic chaperone binding with high affinity to the active sites of certain mutant forms of α-galactosidase, seems to represent an orally alternative to the intravenous administration of ERT for some patients with the amenable form of FD [56].

The effects of ERT administration on GI symptoms and quality of life have been investigated by different research groups, analyzing international registers to which FD patients decided to adhere spontaneously. 

Here, we report three studies: one focused on agalsidase alfa and two on agalsidase beta where patients were interviewed about the persistence of GI symptoms (abdominal pain and diarrhea) after a period of treatment.

The first study by Hoffmann et al. dates back to 2007 [30]. The authors analyzed FD patients enrolled in the Fabry Outcome Survey to investigate changes in GI symptoms and quality of life (using the EQ-5D questionnaire after 12 and 24 months of treatment) with agalsidase alfa, an ERT option. A group of 62 patients (including adult females and males as well as children) was analyzed at baseline and after 12 months of agalsidase alfa. Abdominal pain decreased from 49% (baseline value) to 39% after 12 months of ERT therapy, with a statistically significant improvement in male patients (*p* < 0.05) and in children (*p* < 0.05). Interestingly, both children and men reported the suppression of the abdominal pain for the first time after 12 months of agalsidase alfa. After 24 months, the data on abdominal pain were reported for 58 patients. The prevalence of abdominal pain reduced from 43% (baseline value) to 29% after 24 months of ERT (*p* < 0.05), with a marked reduction in female patients compared to males and children. Diarrhea was investigated in 60 patients. This group included 12 children and 48 adults, of whom 21 females and 39 males. Data were analyzed both at baseline and after 12 months of agalsidase alfa. The only significant result was observed for children, where the symptom decreased from 36% (baseline value) to 7% after 12 months (*p* < 0.05). After 12 months of treatment, diarrhea improved in adults, mainly in males than in females (10% vs. 5%, respectively), although it did not reach statistical significance. After 24 months from the beginning of the agalsidase alpha treatment, data were available for 57 patients (including males, females and children). After 24 months of ERT, diarrhea dropped from 28% (at baseline) to 26%, with a considerable reduction in children from 45% (baseline) to 27%. Thus, in conclusion, 12 months of ERT with agalsidase alfa significantly reduced the prevalence of abdominal pain in male patients and in children. 

Wilcox et al. [57] assessed ERT outcomes with agalsidase beta on GI symptoms in heterozygotes. The authors analyzed data of a cohort of 168 female patients enrolled in the Fabry Registry (NCT00196742; sponsor: Sanofi Genzyme) and self-reporting abdominal pain and/or diarrhoea. Patient received agalsidase beta for at least 2.5 years (average dose of 1.0 mg/kg every 2 weeks). At baseline, abdominal pain and diarrhea were reported by 45% and 39% of females, respectively. At follow-up, the number of women reporting abdominal pain and diarrhea dropped significantly from 45% to 31% (*p* < 0.01) and from 39% to 27% (*p* < 0.01), respectively.

In a recent study, Hopkin et al. [24] investigated the effect on GI symptoms of agalsidase beta in adult males with classic or later-onset FD voluntarily enrolled in The Fabry Registry (NCT00196742, sponsor: Sanofi Genzyme). Abdominal pain and/or diarrhea after a minimum of 0.5 year of follow-up were assessed in eligible male patients receiving an average dose of 1 mg/kg every other week. After a median of 4.7 years of agalsidase beta treatment, the number of classic patients reporting abdominal pain dropped significantly, and after 5.5 years of follow-up the number of patients experiencing episodes of diarrhea also decreased. Conversely, later-onset phenotype male patients reported a reduction of abdominal pain after a median of 4.2 years and diarrhea after a median of 4.4 years of treatment, but results were not statistically significant. Thus, agalsidase beta treatment significantly improved abdominal pain and diarrhea in classic FD male patients and attenuated GI symptoms in later-onset phenotypes.

Hence, ERT with either algasidase alfa or beta provides benefits for the GI system, relieving symptoms and improving quality of life of many FD patients.

## 6. Different Interventions to Attenuate GI symptoms

This section is focused on other interventions besides ERT to treat the GI manifestations. 

Although ERT improves GI manifestation by clearing the GL3 accumulation [22,58], about the 50% of FD patients still complain of GI disorders while on therapy, whereas others develop new GI complications [59]. In case of persisting GI disorders, the available treatments comprise pharmacological treatment, dietary supplements and other strategies such as osteopathy. Drugs represent first-line treatments. Nausea, vomiting, and early satiety are normally treated with metoclopramide, which favors intestinal mobility intestinal mobility [43]; carbamazepine and gabapentin are suggested for the treatment of abdominal pain [60] and loperamide and probiotics have been suggested in the cases of diarrhea [61]. Lai et al. [62] proved that the oral administration of probiotics (i.e., *Lactobacillus casei variety rhamnosus*) administered b.i.d. for seven days to children (n = 81) from 6 months to 6 years old, and hospitalized for acute diarrhea reduced clinical severity and intestinal inflammation, as shown by the reduction of fecal lactoferrin and calprotectin values. Treatment with symptomatic drugs (i.e., antispasmodics) for bloating and abdominal distension, provide relief to patients as well [63,64]. In addition, in a double-blind trial, Bernstein and Kasich demonstrated that simethicone reduces the severity of symptoms such as gas formation and fullness (*p* < 0.001) [65].

Although drugs are useful to solve GI disorders, they do not eradicate the problem. Thus, it is crucial to adopt a correct nutritional style, that can improve the GI status. Despite the fact that there are no existing diets specifically created for the treatment of FD [4], it may be suggested to adopt the same recommendations used for the management of IBS. Namely, foods not recommended for IBS (i.e., caffeine, alcohol, insoluble fiber, fat, spices, and spicy foods) should be avoided in FD. In addition, irregular eating could worsen gut motility; thus, a regular intake of foods is suggested, with no skipping meals and a light dinner at night [66,67]. No studies demonstrate that lactose or gluten can worsen GI symptoms [67]; however, it is suggested that patients with a positive hydrogen breath test should avoid foods containing lactose or gluten.

FD patients could benefit from a diet poor of short-chain fermentable carbohydrates; indeed, these foods attract water in the small intestine and increase gas production, aggravating GI disturbs [68]. In this case, a diet poor of FODMAPS (fermentable carbohydrates or fermentable oligosaccharides, disaccharides, monosaccharides, and polyols) improves bloating, flatulence, diarrhea, and other IBS symptoms [69]. 

Lenders et al. [70] observed a modest improvement in GI symptoms in 7 patients treated with a commercially Orally Delivered Alpha-Galactosidase A source. Patients ingested 600 U orally delivered Alpha-Galactosidase A (total intake: 1800 U) t.i.d. for 90 and 180 days, reporting GI symptoms once a month on a questionnaire provided by the authors. They found a significant decrease on the intensity of abdominal pain (*p* < 0.05), with a reduction of severity of GI symptoms in all patients and a reduction of mild abdominal pain after 12 weeks of treatment in the majority of them. Thus, the authors concluded that oral administered Alpha-Galactosidase A might be a suitable and economical approach for the treatment of GI symptoms in patients affected by FD. However, the authors did not draw any definitive conclusions due to the preliminary results of a limited sample size (n = 7). Further studies are needed to confirm the results.

## 7. Discussion

FD is a chronic, progressive genetic illness characterized by a broad spectrum of manifestations, involving the GI system as well. Frequently, GI symptoms are the earliest and most underestimated signs of FD, as they are often confused with symptoms of IBS or underreported by patients. The most commonly referred GI symptoms are unspecified functional bowel disorders (abdominal cramping and pain, and diarrhea or constipation) typically of IBS [30,71]. Thus, in order to accomplish the diagnosis of FD in the presence of GI manifestations and of typical signs of FD (acroparesthesias, heat and exercise intolerance, corneal opacities, microalbuminuria and angiokeratomas) alpha-galactosidase assay and activity should be determined. Also, questionnaires to score GI symptoms need to be administered to patients with a clinical suspicion of FD [71].

Thus, this approach should favor a prompt FD diagnosis, and ERT should be started before organ damage occurs.

To demonstrate that GI manifestations mark sometimes the beginning of FD, we discussed a clinical case of a female patient carrying a VUS FD genetic mutation and reporting GI symptoms.

We prospectively investigated 85 patients presenting with IBS like symptoms (M/F 29/56, age range 19–80, mean age 46 ± 15 years) referred to our Gastroenterology Outpatient Clinic at SS. Annunziata Hospital, Cento (Ferrara), from February 2018 to July 2019. Alpha-galactosidase plasma activity was assessed in all male patients using fluorometry as a screening test. Should this enzyme be reduced, then plasma globotriaosylsphingosine (lyso-Gb3) was assayed via liquid chromatography tandem mass spectrometry assay. Patients were finally tested for GLA gene mutation analysis using next-generation sequencing by Illumina. All female patients were tested with next-generation sequencing for GLA mutations since first-level screening tests (alpha-galactosidase A and lyso-Gb3 activity) can be frequently normal due to heterozygosity of this X-linked disease. Of the 85 patients enrolled in the study, one female was identified to be a FD carrier. Notably, in this patient, it was detected a class III missense heterozygous mutation of the GLA gene (c.170A> G gene variant p.GIn57Arg) on Exon 1. Gaucher Disease was excluded since β-Glucocerebrosidase activity was normal. The patient turned to have a VUS mutation without typical hallmarks (i.e., renal and cardiac involvement, cornea verticiliata, angiokeratomas, high plasmatic values of lyso-Gb3) of FD and organ damage. Thus, the patient could not be eligible for ERT.

The case we discussed shows the importance of FD in the differential diagnosis of patients suffering from aspecific/IBS-like symptoms. The same Gln57Arg mutation has been identified by Lepedda et al. [72] in four FD patients, all females and none of whom presenting renal involvement; only one out of four was under ERT. Later, the Gln57Arg mutation was found and described as novel by Favalli et al. [73] in a prospective multidisciplinary and multicenter study. Our case report suggests that there is still a lot to learn about different mutations in FD and further studies should be conducted to understand better the VUS FD mutation.

In conclusion, the GI involvement in FD is complex and multifactorial. Digestive symptoms should not be underestimated, as they could contribute to a prompt diagnosis, avoiding the risk of fatal outcomes. In many cases, GI complaints are the first symptoms of the disease, but their underestimation may delay the diagnosis of FD for many years. This complicates the management of FD, while worsening the condition of the patient. General practitioners, internal medicine doctors and gastroenterologists should give adequate attention to the association between GI symptoms and FD. An early FD diagnosis is of paramount importance for the best clinical management of this clinical condition.

## Figures and Tables

**Table 1:** Studies investigating the effect of enzyme replacement therapy (ERT) on gastrointestinal (GI) symptoms.

Author, Year and Reference Number	ERT	GI Symptom Investigated	Months from the First ERT Administration	Number of Investigated Subjects and Gender	Age (Mean ± SD)	Method of Investigation	Outcome
Hoffmann et al., 2007 [30].	Algasidase Alfa.	Abdominal pain.	12	62 FD patients (14 children, 48 adults; 21 females, 41 males).	Age of patients reporting overall GI symptoms Males: 32.3 ± 16.9; Female: 36.9 ± 17.9; Adults: 41.3 ± 13.9; Children 10.9 ± 5.0.	Interviews before and during ERT. Data on HRQoL of patients were collected by EQ-5D questionnaire.	Abdominal pain decreased from 49% (baseline value) to 39%. Both children and men reported a marked improvement of abdominal pain after 12 months of ERT (*p* < 0.05).
			24	58 FD patients (10 children and 48 adults; 25 females, 33 males).			Abdominal pain decreased from 43% (baseline value) to 29 % (*p* < 0.05). Female reported a more significant reduction compared to male patients (from 40% to 20% in females vs. 45% to 36% in males). Also, children reported a reduction of abdominal pain (from 80% at baseline to 50%).
		Diarrhea.	12	60 FD patients (12 children and 48 adults; 21 females and 39 males).			Diarrhea decreased notably only in children, from 36% at the baseline to 7% after 12 months of ERT (*p* < 0.05).
			24	57 FD patients (11 children and 48 adults; 25 females and 32 males).			Diarrhea significantly improved in children (from 45% of symptom reported at baseline to 27% after 24 months).
Wilcox et al., 2018 [57].	Agalsidase Beta.	Abdominal pain.	The observation interval started with the first ERT treatment and ended when one of the following occurred: (1) discontinuation of agalsidase beta treatment; or (2) switching of agalsidase beta treatment to another treatment.	168 FD females patients.	Data describing abdominal pain were available for 166 patients, aged 43.3 ± 14.5 years at first ERT administration.	Self-reported GI symptoms at last clinical visit compared to treatment-baseline.	The number of women reporting abdominal pain and diarrhea dropped significantly from 45% (at baseline) to 31% at last follow-up (*p* < 0.01).
		Diarrhea.			Data describing diarrhea were available for 160 patients, aged 43.1 ± 14.6 years at first ERT administration.		Diarrhea improved from 39% (baseline value) to 27% at the last follow-up (*p* < 0.01).
Hopkin et al., 2020 [24].	Agalsidase Beta.	Abdominal pain.	Median of 4.7 years of treatment.	171 classic phenotype FD male patients.	Data for abdominal pain were available for 171 classic phenotype FD male patients, aged 36.2 years (28.9–43.7) at first ERT administration.	Self-reported GI symptoms at last clinical visit (≥0.5 year of follow-up) compared to treatment-baseline.	The number of classic patients with abdominal pain dropped significantly: 96 out of 171 (56%) vs. 70 out of 171 (41%) (*p* < 0.001).
		Diarrhea.	5.5 years of follow-up.		Data describing diarrhea were available for 169 classic phenotype FD male patients, aged 35.8 years (27.9–43.3) at first ERT administration (in brackets the 25th–75th percentile values).		The number of patients reporting episodes of diarrhea decreased significantly: 97 out of 169, (57%) vs. 80 out 169 (47%) (*p* < 0.05).

Note: ERT, enzyme replacement therapy; FD, Fabry Disease; HRQoL, Health-Related Quality of Life; EQ-5 D, EuroQol- 5 Dimension questionnaire; SD, Standard Deviation.
